# The impact of public responses toward healthcare workers on their work engagement and well-being during the Covid-19 pandemic

**DOI:** 10.3389/fpsyg.2022.949153

**Published:** 2022-12-01

**Authors:** Wen Shan, Zhengkui Wang, Millie Yun Su

**Affiliations:** ^1^Human Resource Management, S R Nathan School of Human Development, Singapore University of Social Science, Singapore, Singapore; ^2^InfoComm Technology Cluster, Singapore Institute of Technology, Singapore, Singapore

**Keywords:** COVID-19 pandemic, healthcare workers, public response, work engagement, well-being, sentiment analysis, topic modeling

## Abstract

**Introduction/context:**

Healthcare workers (HCWs) play an important role in fighting against the COVID-19 pandemic. However, they have been exposed to mixed public responses more significantly during the COVID-19 pandemic, which have potentially affected their work and life.

**Aim:**

We aim to study what public responses toward HCWs existed, how and why such public responses impacted HCW’s work engagement and well-being, and how Human Resource (HR) professionals navigate these impacts. These understandings are important for improving HCWs’ work and life quality.

**Methods:**

We adopted a mixed approach including both quantitative and qualitative methods to investigate how the public responses impact HCWs’ work engagement and well-being and how human resource management (HRM) shall intervene. Our quantitative study enables us to collect and analyze a large amount of public responses toward HCWs from the social media platform during the COVID-19 pandemic globally, and uncover the sentiments and topics of these pubic responses *via* big data and AI technologies. Our qualitative study allows us to understand how and why these public responses impact HCWs’ work engagement and well-being *via* interviews and further identify how HR professionals shall navigate these impacts.

**Results:**

The sentiment analysis showed that 55.9% of the discussions toward HCWs were positive, 27.2% were neutral, and 16.9% were negative. The topic modeling analysis indicated that the commonly identified topics were related to fear (the negative responses) and gratitude (the positive responses). The interviews with 18 HCWs revealed that HCWs’ work engagement and well-being were decreased by negative public responses through experiencing tension or disappointment due to social and physical ostracism, rejection, discrimination, and criticism. On the other hand, positive public responses in terms of encouragement, recognition, and tangible donations increased their work engagement and well-being. The analysis also suggested that occupational calling served as a mechanism that explained why public responses had such impacts on HCWs. The interview results also highlighted the significance of HRM in bridging positive public responses toward HCWs and revealed problems with communication from HRM during the pandemic. This research provides practical implications about how to improve HCWs work engagement and well-being during the pandemic *via* public and HRM efforts.

## Introduction

The COVID-19 pandemic has brought about significant disruptions to people’s daily lives globally, especially to the lives of employees working in the healthcare industry (WHO, 2021). Healthcare Workers (HCWs) are at the center stage in tackling this public health crisis as a global threat. Many of them are frontline workers working in proximity to patients who may be exposed to close contact with the virus. They are the ones who sacrifice their personal and family time carrying out their roles in hospitals to take care of numerous patients, including COVID-19 patients. Undoubtedly, the COVID-19 pandemic has brought tremendous stress on the healthcare system and healthcare professionals (e.g., Tan et al., 2020a). The stress has been caused by long working hours, night shifts, a large volume of patients, constant exposure to perceived risk, constant change in protocols, and the lack of facilities, equipment, and manpower (e.g., Hwang et al., 2020; Khanal et al., 2020; Pappa et al., 2020).

Besides coping with the physical, mental and emotional stress, HCWs may also be exposed to being stigmatized, discriminated against, and ostracized by the public in the workplace and surroundings (Bagcchi, 2020; Singh and Subedi, 2020). For example, in various countries, HCWs were reportedly denied access to public transport, insulted in the street, and even evicted from rented apartments (Bagcchi, 2020). HCWs also experienced gossip and bullying in communities in various countries (Dye et al., 2020). These negative public responses could make them more vulnerable to negative psychological consequences (Xiong and Peng, 2020). These problems may distract their attention and decision-making abilities, posing a threat to their work engagement and well-being, which might also affect their ability to manage crises or be exposed to occupational hazards.

At the same time, the COVID-19 pandemic has also highlighted the importance of healthcare professionals due to the increase in public awareness and recognition. HCWs were essential workers and have become labeled as heroes during the COVID-19 pandemic (Booth et al., 2020). To recognize their efforts, people have shown their appreciation online by posting pictures of appreciation (Wicker, 2020) and mural paintings on social media and through material provisions like food donations, airline tickets, and vouchers (Allen, 2020; Hessekiel, 2020). Meanwhile, the public responses may also impact HCWs in different ways. For example, a study conducted in Africa found that the pandemic had strengthened their sense of duty and their role as a nurse due to the reinforced feeling of pride and appreciation from the community for their contribution (Marey-Sarwan et al., 2022). A similar phenomenon took place during the SARS epidemics where HCWs in Singapore experienced social stigmatization (49%), while most (77%) felt appreciated by society and heartened by the social support they received (Koh et al., 2005).

As such, existing studies have suggested both positive and negative impacts of public views toward HCWs during times of public health crisis. However, these studies have not aggregated public responses systematically. In other words, we know little about what was being said in the public response toward HCWs during the health crisis, and we also have limited understanding of how and why the HCWs perceived public responses impact their work life, particularly their engagement and well-being.

As the International Committee of The Red Cross appeals, “the recent displays of public support for COVID-19 responders are heartwarming, but many responders are nevertheless experiencing harassment, stigmatization, and physical violence. Health personnel, medical facilities, and transport such as ambulances must be respected and protected in all circumstances, and the work of medical personnel must be facilitated at all times (IFMSA, 2020, pp.1).” To answer this call, it is imperative for scholars to examine public responses toward HCWs on a large scale during the COVID-19 pandemic, and how they impact HCWs’ work engagement and well-being. As long as effective HRM initiatives and practices are implemented, they can improve HCWs’ work engagement and well-being (Alfes et al., 2012; Shantz et al., 2016). Therefore, in this paper, our research aims to better understand public responses toward HCWs *via* social media analysis by employing AI and big data technologies, and identify the major types of topics and sentiments under each type of response. More importantly, our research aims to reveal the impacts of such public responses on HCWs’ work engagement and well-being during the COVID-19 pandemic and offers valuable insights for HRM professionals to foster engagement and well-being in the healthcare industry. This, in turn, may ensure a more sustainable workforce that can deliver high-quality work during public health crises.

## Literature review

### Public responses and their impacts on HCWs

During the time of the public health crisis, many people rely on the media to obtain relevant information. Due to the prevalent use of social media, the public is also free to share opinions and views, which formulates the public response. As limited studies have suggested that media exposure could induce stress (e.g., Garfin et al., 2020; Secker and Braithwaite, 2021), there is also a lack of studies that examine public response as a factor to occupational stress, which would also affect work engagement and well-being. However, we can refer to some indirect evidence from existing research. For example, when the public responses are positive, HCWs may interpret them as a form of social support, which has been proved to be inversely related to depression, anxiety, irritability, sleep quality, and loneliness during the COVID-19 pandemic (Grey et al., 2020; Cohen and Nica, 2021). Being recognized and appreciated by the organization at the workplace was also found to improve work engagement and well-being among HCWs (Strömgren et al., 2016; Anwar and Qadir, 2017; Denning et al., 2021). This indirect evidence posits the proposition that positive public responses may enhance HCWs’ work engagement and well-being. On the other hand, the negative responses in public are assumed to decrease HCWs’ work engagement and well-being during the pandemic.

### HCW’s work engagement

Employee work engagement is defined as an individual’s level of commitment to and involvement in their organization and its goals in general (Markos and Sridevi, 2010). Opposite to burnout (González-Romá et al., 2006), work engagement entails vigor, dedication, and absorption in one’s work (Schaufeli et al., 2002). During the COVID-19 pandemic, work engagement among HCWs served as a type of mental resilience and a protective strategy against burnout (Allande-Cussó et al., 2021). Its importance also manifests in its direct prediction of job performance (Christian et al., 2011), employee retention, and safety (Harter et al., 2002).

Research showed mixed impacts of COVID-19 on HCWs’ work engagement (Nemțeanu et al., 2022). In some studies, most HCW participants self-reported symptoms of burnout in Singapore (Lum et al., 2021), Italy (Barello et al., 2020), and other countries (Denning et al., 2021). On the contrary, in China and Spain, frontline HCWs were found to have high levels of work engagement, such as dedication (Gómez-Salgado et al., 2021; Zhang et al., 2021). This implies that there are other understudied factors that may impact HCWs’ work engagement during the COVID-19 pandemic.

### HCW’s well-being

Well-being at the workplace includes the physical, psychological, and emotional health of employees in terms of their overall state of being comfortable, healthy, and happy (Pradhan and Hati, 2019). HCWs’ well-being is significantly more important compared to other industries due to the grave challenges that they face at their workplace (Tomo and De Simone, 2017). Such challenges included difficult work schedules such as night shift work, being exposed to infectious diseases, and psychological stressors, which negatively affected their well-being (Tomo and De Simone, 2017). These workplace conditions have been aggravated by the COVID-19 pandemic, which has badly affected HCW’s well-being (Denning et al., 2021; San Juan et al., 2021). In the last 2 years, HCWs have experienced burnout, work-family imbalance, and high levels of anxiety, depression, fear, and stress (Marton et al., 2020; Tan et al., 2020b). These work conditions may be hard to change due to the variants of viruses, but other factors might be the key to improving HCWs’ well-being.

### HCW’s calling

For many HCWs, their career choice was a calling in terms of making a difference in society and helping others (Gordon and Nelson, 2005). Individuals with career or occupational calling work with a strong sense of meaning and purpose and have a desire to contribute to the community (Dik and Duffy, 2009; Elangovan et al., 2010). It has also been established that perceived calling relates to a higher level of work engagement (Duffy et al., 2011; Hirschi, 2012; Ziedelis, 2019). As such, occupational calling served as a psychological buffer for HCWs in challenging situations, such that stronger occupational calling weakened the association between stressors and burnout among healthcare professionals (Creed et al., 2014). As a result, individuals with a realized occupational calling would have higher work engagement and healthier well-being than those who had no calling at all (Gazica and Spector, 2015). On the other hand, when individuals with clear occupational callings fail to perform these callings (Berg et al., 2010), they often show lower work engagement and well-being than those who are living their perceived calling or who do not have a calling to a particular vocation (Gazica and Spector, 2015).

### Self-determination theory

We contend that HCW’s occupational calling may serve as a mechanism to explain why public responses impact their work engagement and well-being. As the theoretical foundation, we draw upon the Self-Determination Theory (SDT; Ryan and Deci, 2000). Essentially, the SDT assumes that people are motivated to grow, master, and integrate experiences into a holistic sense of self on three bases of needs—the need for autonomy, competence, and relatedness. And the development of the sense of self requires individuals’ ongoing interactions with the social environments surrounding them. These social environments can either support or inhibit these needs. This dynamic of individuals socializing with the environment shapes the predictions about people’s work, life experiences, and psychological states twofold. First, the ongoing satisfaction of these three needs facilitates well-being and work engagement. On the other hand, if some or even all these needs are unmet, there will be detrimental effects on individuals’ work engagement and well-being (Ryan and Deci, 2000).

During the COVID-19 pandemic, public responses are one important factor in the social environment that impacts HCW’s three needs altogether. Based on self-determination theory, healthcare workers tend to be intrinsically motivated with the utmost desire to help others (Muthuri et al., 2020), which entails relatedness (i.e., the need to feel connected with others), competence (i.e., the need to feel effective in achieving desired outcomes), and autonomy (i.e., the urge to act consistent with an integrated sense of self and take direct actions that would result in real change; Gagné and Deci, 2005; Broeck et al., 2010). As such, being recognized and appreciated by the public can satisfy HCWs’ basic psychological needs, which improve their work engagement and positive well-being (Gazica and Spector, 2015). On the other hand, negative public responses in the social environments would bring adverse effects on HCWs’ work engagement and well-being. Their need for relatedness is not satisfied because they are ostracized by the people whom they are called to help and thus end up feeling discouraged or disheartened. Their need for autonomy is compromised because they may feel that they are not able to control public responses, including discrimination and stereotypes. Their need for competence is also unfulfilled because they may see that the public does not accept their suggestions or acknowledge their contribution and thus end up feeling helpless or useless in performing their roles. In these ways, both positive and negative public responses can significantly impact HCWs’ work engagement and well-being during the COVID-19 pandemic.

Nonetheless, not all HCWs are motivated by calling. There are other HCWs who are motivated by extrinsic factors, such as money and job security (Muthuri et al., 2020). In this case, their relatedness, competence, and autonomy needs might not be associated with their careers. Thus, we propose that public responses have more impact on the calling-motivated HCWs than on the extrinsically-motivated HCWs.

## Overview of studies

To uncover public responses toward HCWs during the COVID-19 pandemic, how and why they impact HCWs’ work engagement and well-being, and how HRM can navigate such impacts, we conducted two studies with both quantitative and qualitative methods. Study 1, as a quantitative study, focuses on automatically understanding the public responses toward HCWs by analyzing the large scale of social media data *via* AI and big data analytics. Adopting AI and big data technologies to understand the HCWs’ work engagement and well-being has been promising for collecting public information (Campbell and Popescu, 2021; Maxwell and Grupac, 2021; Nemțeanu et al., 2021; Riley and Nica, 2021). In order to obtain the public responses, we developed automatic data scripters to crawl the Tweets toward HCWs from the Twitter platform during the COVID-19 pandemic. Based on the crawled tweets, we performed the sentiment analysis to discover the sentiments or emotions (i.e., positive, negative, or neutral) for each tweet toward HCWs. Furthermore, we conducted the topic modeling to analyze what are the topics or concerns for each category of tweets. This enables us to understand their emotions and concerns toward HCWs.

Study 2, as a qualitative study, further examined how and why such mixed public responses impacted HCWs’ work engagement and well-being in different ways. We interviewed 18 HCWs working in Singapore and asked about their calling to be HCWs, the factors that impacted their work engagement and well-being during the COVID-19 pandemic, and what their HRM did effectively or ineffectively. We transcribed their responses for qualitative coding, based on which we identified how positive and negative public responses influenced HCWs’ work engagement and well-being and effective HRM practices. We also further compared the impacts of public responses on those who had an intrinsic calling versus those who did not have an intrinsic calling.

## Study 1 on public responses *via* big data

### Materials and methods

Social media platforms (e.g., Twitter and Facebook) have been widely used for people to share their opinions and thoughts. Twitter, as one of the world’s largest social network platforms, hosts a plethora of user-generated posts that closely reflect public reactions (Szomszor et al., 2009). Therefore, we used Twitter as our data source to collect the public responses toward HCWs. This enables us to get responses from a large number of users, which are more representative of the public. Our method consists of several major components, including data collection, data cleaning and pre-processing, sentiment analysis, and topic detection under each sentiment. The details of each component are provided below.

### Data collection

The objective of data collection is to collect public responses toward HCWs during the COVID-19 pandemic from Twitter. To do so, we developed one automated data scripter using Python to crawl the tweets that related to HCWs. The tweets were crawled using a certain set of hashtags related to HCWs, such as #healthcareworkers, #nurses, #frontlineworkers, #doctors, #essential, #firstresponders, #publichealth, #thankyoufrontliners, #nursesrock, #frontliners, #healthcareprofessional, #savinglives, #fightCOVID-19, #worldhealthday, #thankyoufrontlineworkers, and so on. These hashtags are the popular ones related to HCWs. Therefore, when the discussion includes these hashtags, the tweets are most likely talking about something related to HCWs. The crawler scraped 100 tweets at a 15-min interval. In the end, we crawled 400,000 tweets related to HCWs during the COVID-19 pandemic.

#### Data cleaning and pre-processing

After crawling the data, we first cleaned the dataset such that the irrelevant information in the data would be removed to improve the analysis performance. Irrelevant information mainly includes those irrelevant symbols or characters that exist in the tweets but may not be needed in the analysis. Below are the major data-cleaning tasks performed to clean the tweets.

##### Removing NaN values

For some tweets, there were NaN values that do not contribute to the meaning of the data. The NaN stands for Not A Number and is normally used to represent the missing value in the data. Thus, all the NaN values were removed.

##### Removing duplicate tweets

Duplicated tweets were also removed as duplicate data might affect the analysis to cause some form of extreme sampling or bias to the model.

##### Removing tweets with less than three words

We also removed all the very short tweets (e.g., containing less than three words), as they may not be meaningful and effective enough for the analysis.

##### Removing @users

Usernames are not relevant and are noises for the analysis. Thus, they were all removed before the analysis.

##### Removing URLs

URLs in tweets are not useful as they do not contain words of sentimental value or topics, which were thus removed.

##### Removing special characters

Special characters refer to the irrelevant symbols (e.g., #, **!,** $, etc.), and numbers that do not contribute to the analysis. These characters were removed.

##### Removing RT

Tweets that were retweeted from another tweet have the “RT” in them. This does not contribute any meaning to the analysis, and they were removed as well.

#### Removing stop words

Stop words refer to the most common words in a language that does not add much meaning to a sentence, such as “the,” “an,” “so,” “the,” and “what.” These words can be safely ignored without sacrificing the meaning of the sentence. As this information does not provide any information to our model, they were also removed. We obtained the stop words list from the Natural Language Toolkit (NLTK).^1^

##### Tokenization

Tokenization is another most common task in natural language processing to pre-process text data. It is essentially splitting a tweet (e.g., phrase, sentence, or paragraph) into smaller units, such as individual words or terms. Each of these smaller units is called a token. Tokenization helps us to identify the words that constitute a string of characters. This is an important step, as the meaning of the text could be easily interpreted by analyzing the words present in the text.

Lemmatization. For grammatical reasons, the sentences in human language may use different forms of a word (e.g., are, were, going) or families of derivationally related words with similar meanings (e.g., democracy, democratic, and democratization). However, these words may refer to the same meaning. Therefore, we performed the lemmatization to reduce inflectional forms and derivationally related forms of a word to a common base form, which is used to build the dictionary, models, and evaluations.

#### Sentiment analysis

To understand the emotions of the responses toward HCWs and associated topics, we conducted sentiment analysis on the processed dataset to detect and categorize the tweets based on their sentiment. We categorized all the tweets into three emotion categories, positive, negative, and neutral.

To conduct sentimental analysis, we employed the Natural Language Toolkit (NLTK), which can process the natural human language data to provide statistical natural language insights. VADER (Valence Aware Dictionary for Sentiment Reasoning) was chosen for the analysis as it does not require any prior training data and can easily understand text even if there are punctuations and common speech text (Rahul et al., 2021). Based on VADER, we evaluated the sentiment scores (also referred to as the polarity scores) for each tweet. The polarity score returned from using VADER can be categorized into the categories “negative,” “neutral,” “positive,” and “compound.” Each category refers to the sentiment scores of the tweet, with the compound category representing the sentence’s emotion. For example, a tweet with the score of {“neg:” 0.539, “neu:” 0.261, “pos:” 0.0, “compound:” −0.8849} could represent that the tweet has mostly negative and neutral sentiments and no positive sentiments at all. The “compound” key, which is calculated by normalizing the other three emotions, represents the overall sentiment of the sentence. If the compound value is more than 0.05, the analyzed tweet is positive. If it is less than −0.05, it is negative. Otherwise, it is neutral.

#### Topic detection

After the sentiment analysis, we also studied, in each category of the emotional tweets, what topics were mentioned. This helps us better understand the reason why they had such emotion while they commented toward HCWs on Twitter.

To do so, we employed topic modeling techniques to automatically identify the topics embedded in the tweets. Topic modeling is the task of using unsupervised learning to extract the main topics (represented as a set of words) that occur in a collection of documents like tweets (Wang et al., 2017). We first divided all the tweets into three categories (i.e., positive, negative, and neutral) according to their sentiments. For each category of tweets, we built an LDA (Latent Dirichlet Allocation) model using Gensim AI libraries to extract the topics under the collection of tweets. Gensim was chosen as it is easy to build topic modeling (Akef et al., 2016). The only concern is that clean data are required to be in the form of tokenized words. After the data are pre-processed, a dictionary and corpus need to be created to be used as input for LDA models. Gensim Corpora Dictionary was created to store the mapping of words and their integer IDs. To generate the Corpus, Gensim Corpora Dictionary doc2bow was used. The corpus was then mapped to the words found in the lemmatized parsed data and its frequency.

To determine if the topic modeling is good, we evaluate it based on the result of the coherence score and its compute perplexity. A higher value in coherence score or a lower value in compute complexity represents a better topic modeling approach.

### Results

#### Sentiment analysis

To evaluate the overall sentiments of the dataset, we made use of the Matplotlib library to better visualize the output of the sentiment scores. The result indicates that most of the tweets concerning HCWs were of positive sentiments. In the entire dataset, 55.9%, 27.2%, and 16.9% of them were positive, neutral, and negative, respectively. This shows that tweets that mention HCWs were generally positive in nature, with a small portion of them being negative.

**Figure 1:**
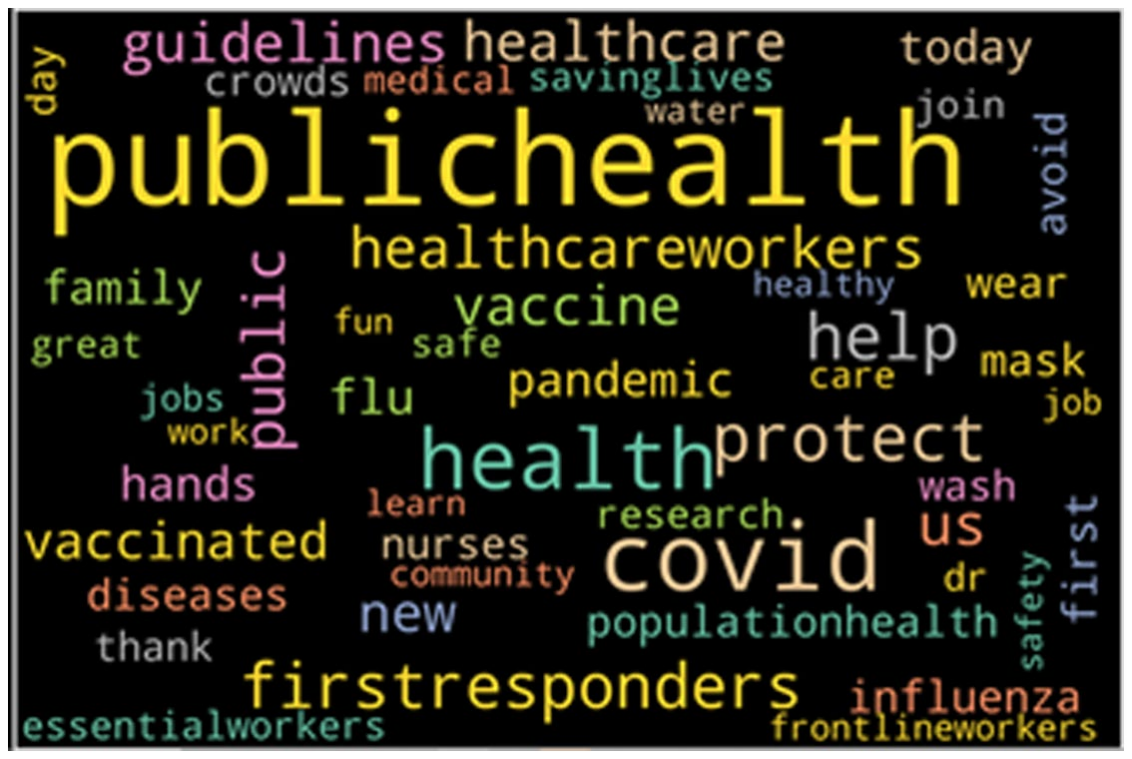
The topic 50 keywords from the entire dataset.

Furthermore, using word clouds to visualize, we can dive deeper into what words were used frequently in regard to the various sentiments. Using both the Matplotlib and word cloud library, we visualized the most 50 frequent words grouped by their sentiments. Figure 1 provides an overview of the overall word cloud with the top 50 most frequently mentioned words in the entire dataset. The bigger the font size of the word, the more frequently it is mentioned. From the result, we can see that most of the words used are not negative in nature.

**Figure 2:**
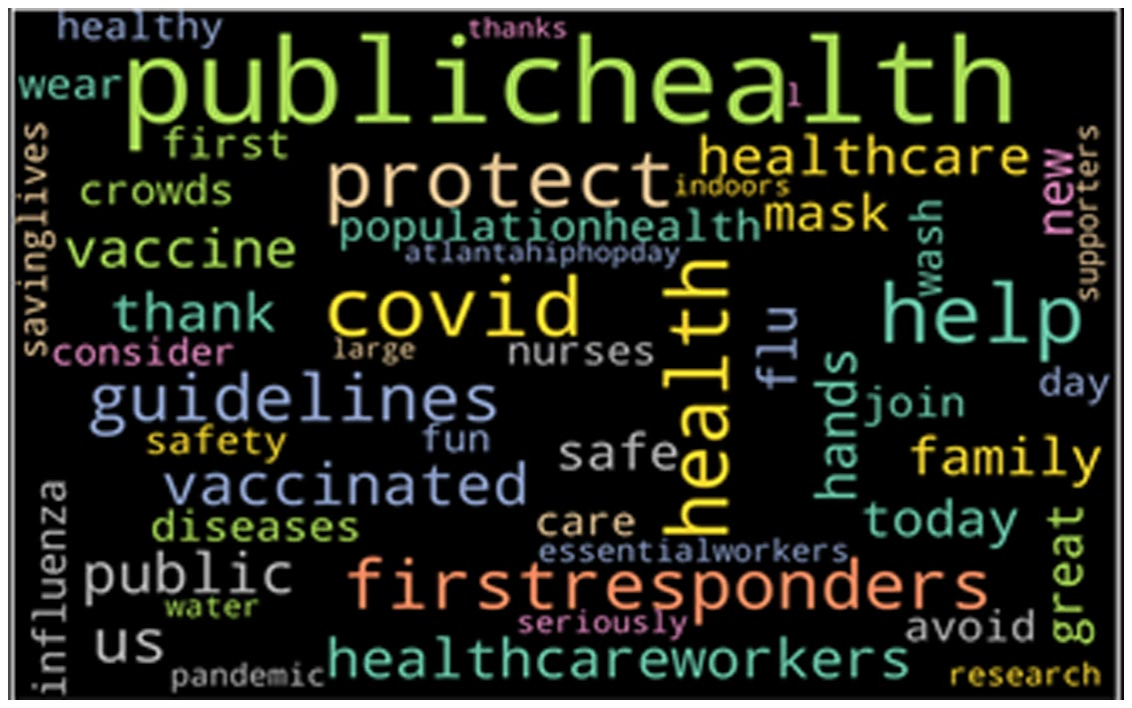
The top 50 keywords from positive tweets.

Figure 2 provides the word cloud with the top 50 most frequently mentioned words of tweets that have a positive sentiment. From the word cloud, we can see that when people discuss toward HCWs, many words like “protect,” “help,” “thank,” and “health” are used frequently. These are all words of gratitude toward the HCWs, including appreciation and positive wishes.

**Figure 3:**
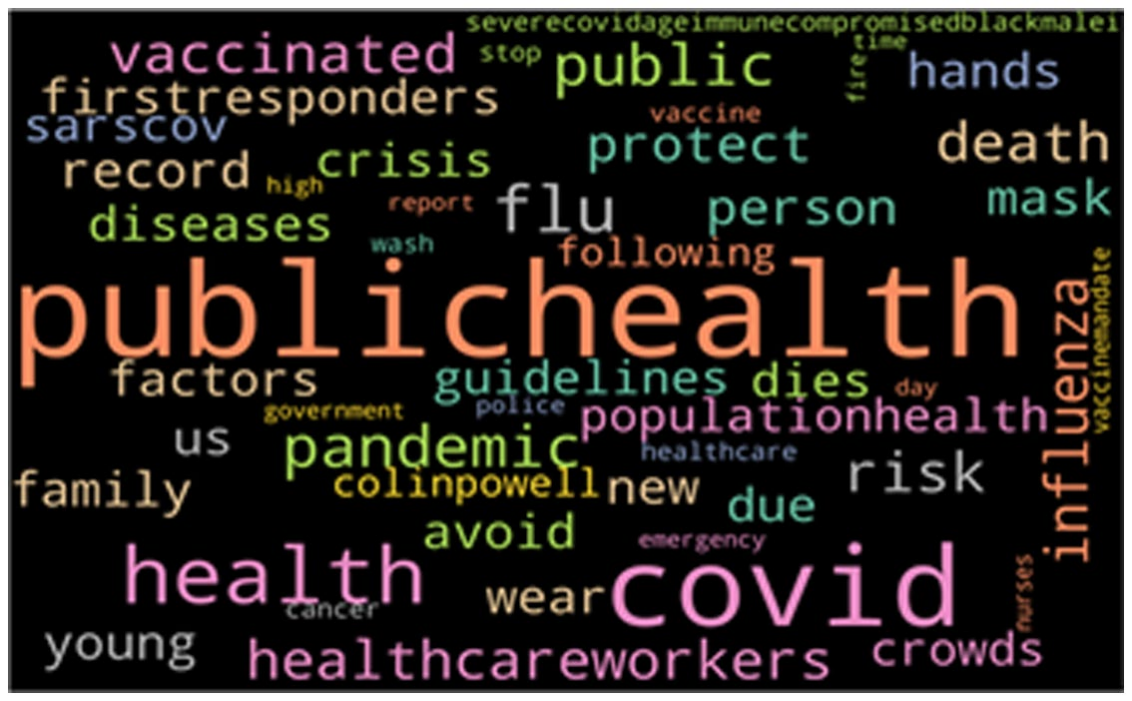
The top 50 keywords from negative tweets.

Figure 3 indicates the negative word cloud with the top 50 most frequently mentioned words of tweets that have a negative sentiment. As seen, many words (e.g., “death,” “stop,” “dies,” “crowds,” and “crisis”) are frequently used in such tweets that could be inferred as pessimistic words to use. These are all words of fear and uncertainty toward the HCWs, implying rejection and stereotypes.

**Figure 4:**
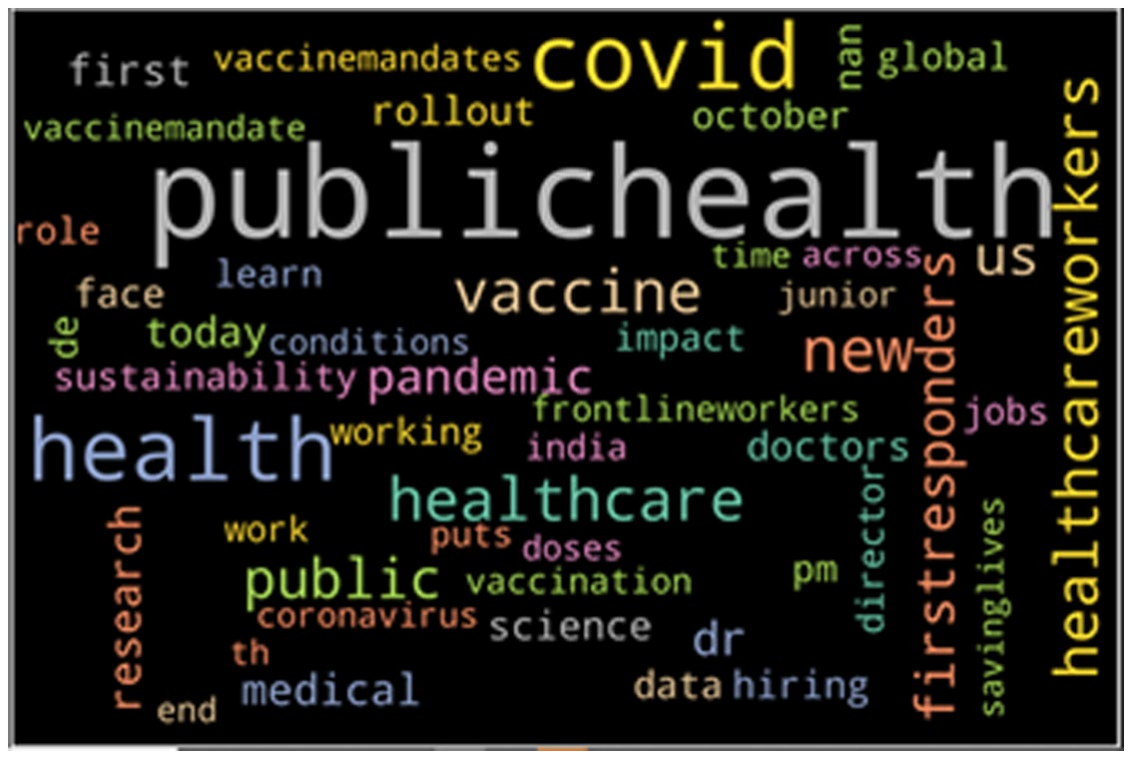
The top 50 keywords from the neutral tweets.

Lastly, Figure 4 provides the word cloud that shows the top 50 most frequently mentioned words of tweets with a neutral sentiment. As seen from the word cloud, when people discuss toward HCWs neutrally, they use more neutral words. Many words like “science,” “doctors,” “medical,” and “research” are frequently used in such tweets.

#### Subjectivity analysis

In addition, we also studied the subjectivity and objectivity of the tweets. Subjective sentences generally refer to personal opinion, emotion, or judgment, whereas objective refers to factual information. To conduct the subjectivity analysis, we utilized Textblob,^2^ which is a python API commonly used in natural language processing. Using Textblob, we identified the subjectivity and objectivity of each tweet. Our finding shows that 72.5% of tweets are objective, and 27.5% are subjective. Since objective tweets are tweets that are more rational and unbiased, we can conclude that majority of tweets and comments toward HCWs on Twitter are not influenced by the person’s emotion or judgment but by factual information.

##### Subjectivity vs. polarity

**Figure 5:**
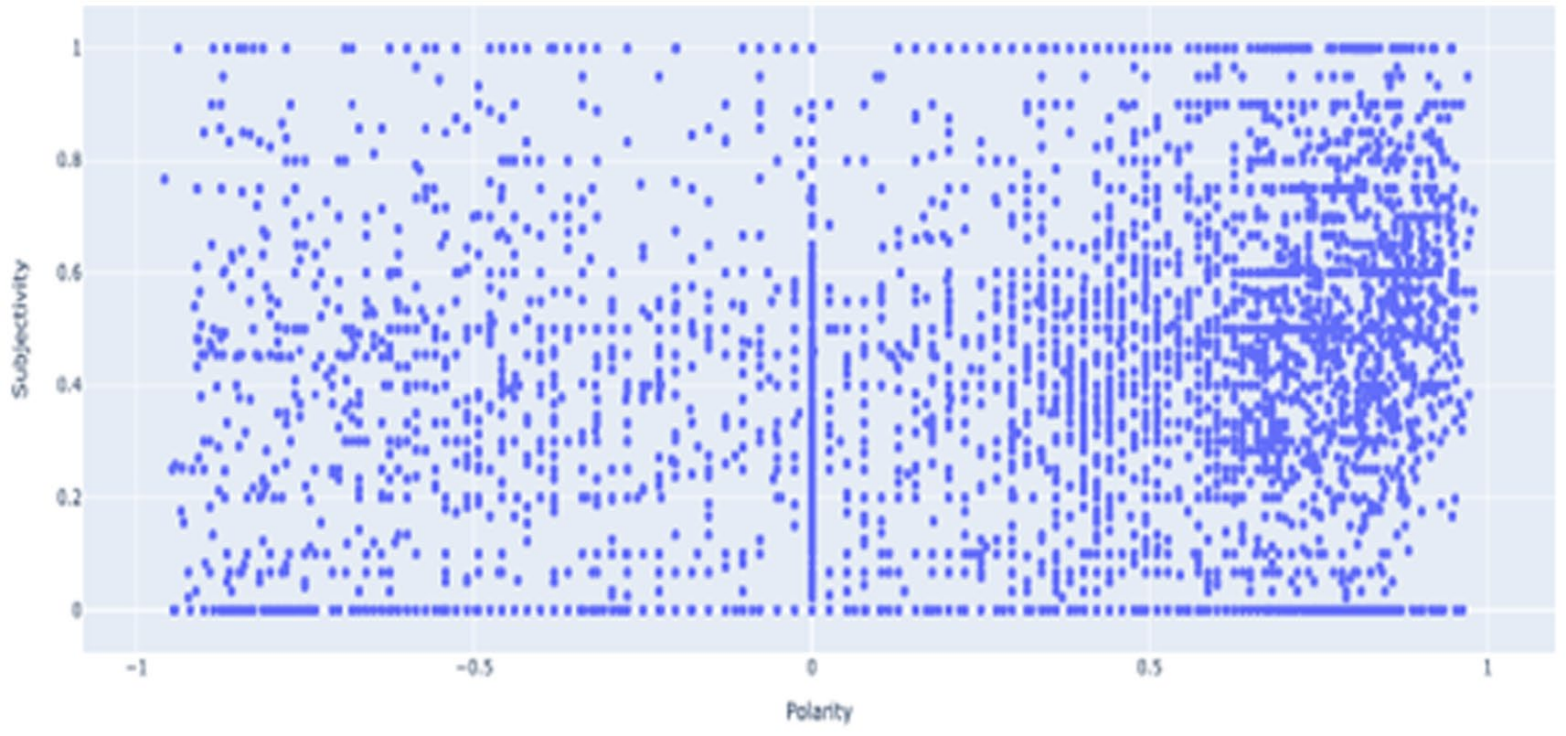
Subjectivity vs. polarity scatterplot.

To compare subjectivity and polarity, we provide a scatter plot as shown in Figure 5 using Ploty Express. As shown in the scatter plot, there is a cluster around polarity from 0.6 to 0.8 and subjectivity from 0.2 to 0.5. This shows that most of the tweets are positive in polarity and objective. This indicates that most of the tweets are rational and of positive sentiments toward the HCWs during the COVID-19 pandemic.

##### Topic detection

**Figure 6:**
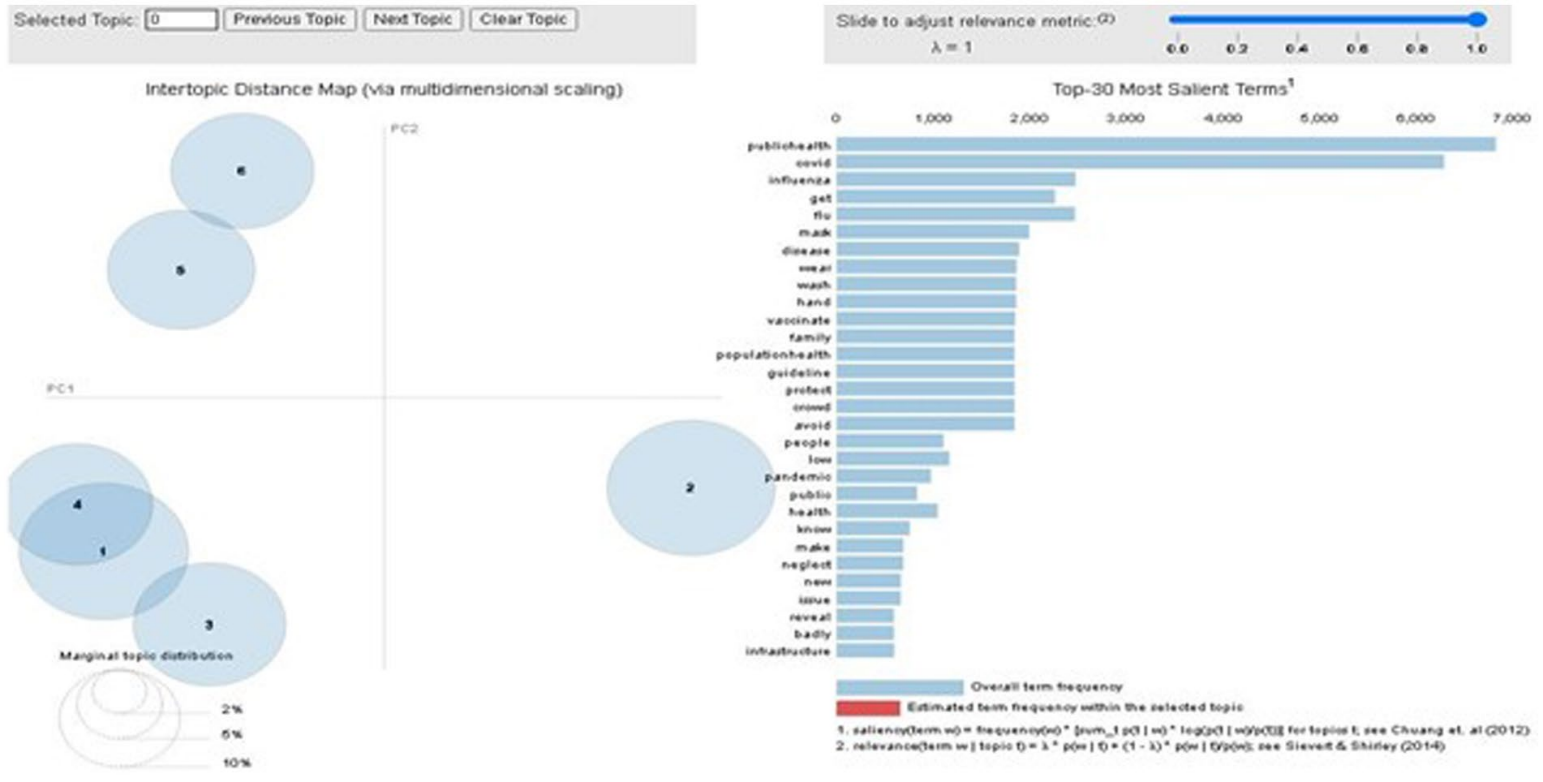
Negative topic words in Gensim LDA model.

Next, we study the topics under each category of sentiment tweets. To be intuitive, we used an interactive chart to display the results using PyLDAvis^3^ such that the topics could be easily interpreted. Figure 6 shows the identified results for the negative tweets. The left side of the six bubbles indicates six identified topics. While clicking each topic on the left, the right side shows all the words included in the topics, where the bar size represents the overall frequency of the words that appeared in this topic.

**Figure 7:**
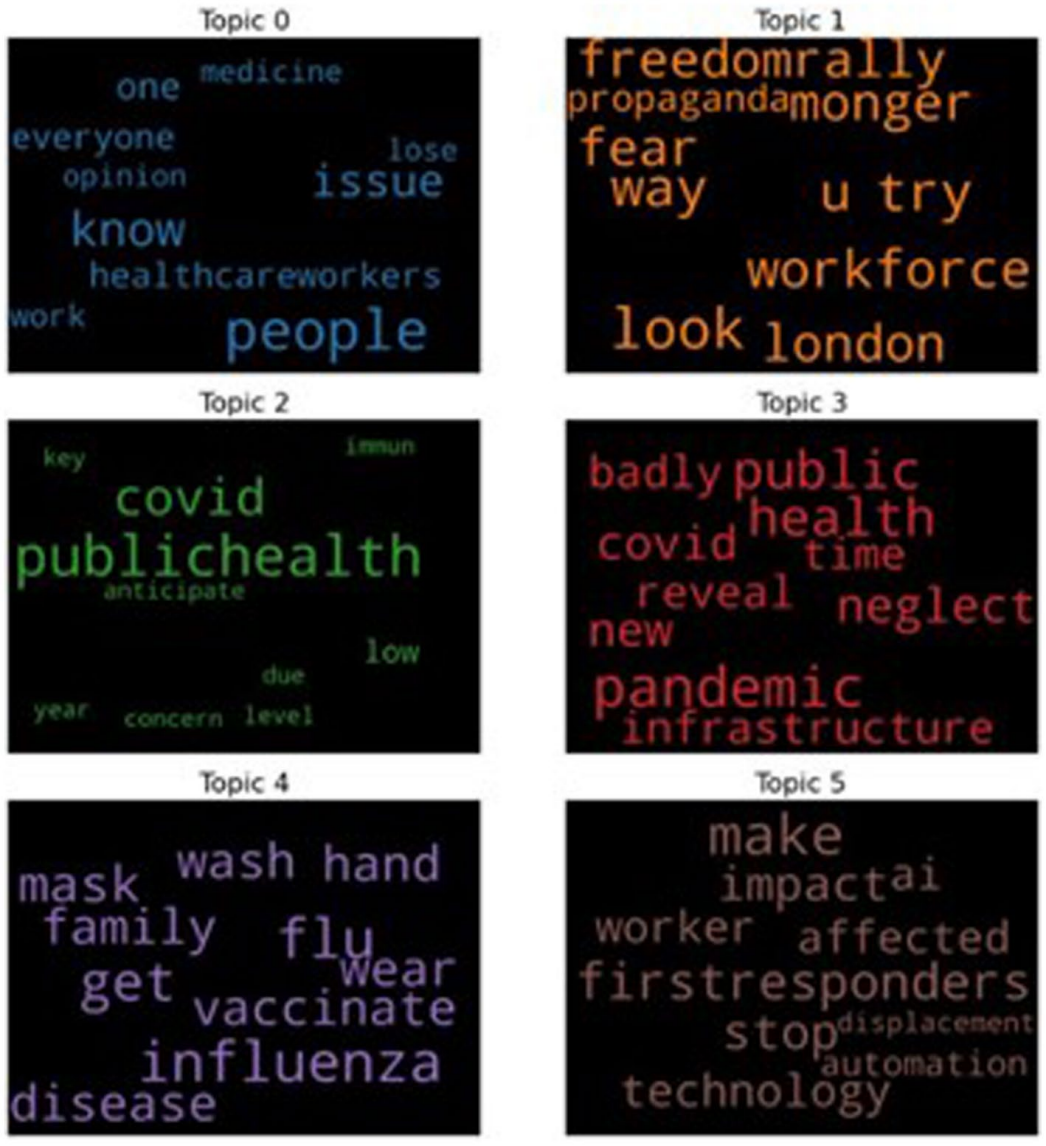
The top six topics for the tweets with the negative sentiments.

As the image result (as in Figure 6) in Gensim LDA model cannot be fully visualized, for ease of presentation, we also present the six topics with the involved top 10 keywords into the word cloud presentations, as shown in Figure 7. From the keywords in each topic, we can roughly identify the major meaning of each topic for those tweets with negative sentiments, which are provided below:

Topic 0: related to uncertainty.
Topic 1: related to politics.
Topic 2: related to COVID-19.
Topic 3: related to pandemic.
Topic 4: related to precaution measures.
Topic 5: related to economy and working practice.

Figure 7 shows that when people had negative emotions in tweets, the top six topics mentioned were related to uncertainty, politics, COVID-19, pandemic, precaution measures, economy, and working practices. In particular, most of the tweets revolved around the idea of being unsure of how the pandemic is going to be and how the disease will affect the people infected, especially the HCWs. There were also concerns about how it would affect the economy and what kind of response should be taken in current situations.

**Figure 8:**
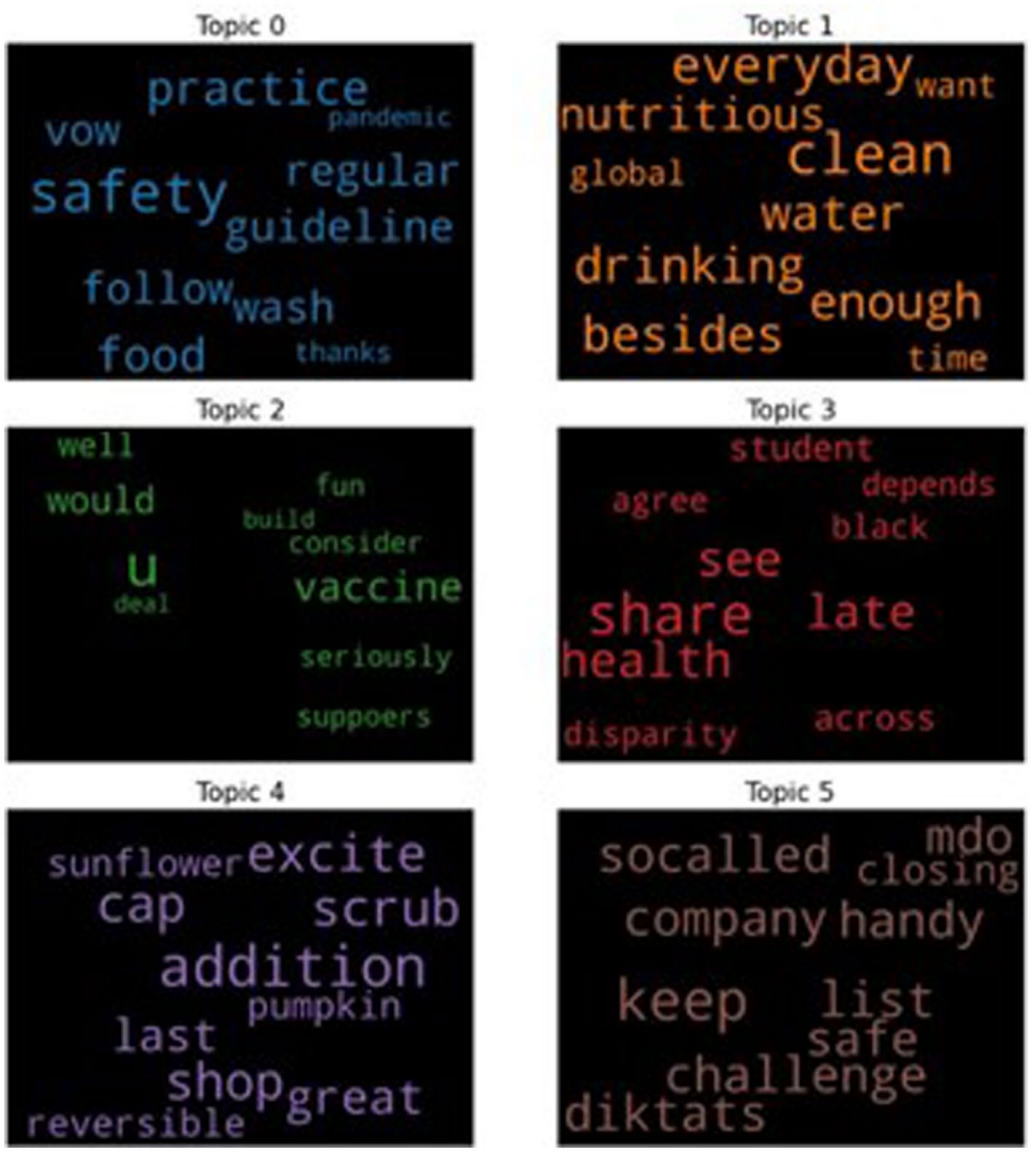
The top six topics for the tweets with the positive sentiments.

For all the tweets with positive sentiments, we also performed the topic modeling, and the results are shown in the interactive chart in Figure 6. However, as the interactive chart cannot be fully displayed in figures, we will only show the identified top six topics, each of which is shown in a word cloud with the top 10 keywords in Figure 8.

From Figure 8, we can see that the following are the possible topics that are appearing in each word cloud based on all the positive tweets:

Topic 0: related to good practices.
Topic 1: related to lifestyles.
Topic 2: related to vaccines.
Topic 3: related to health.
Topic 4: related to nutritious food.
Topic 5: related to safety measurements for the company.

The result indicates that when people were tweeting positively, the tweets likely discussed good practices, healthy lifestyles, vaccines, health, nutritious food, and safe measurements for the company. On the positive word clouds, tweets include ideas on how to prevent infection, maintain good health, and shop for nutritious food like sunflower and pumpkin, and company measures to keep people safe. Such words can be considered caring and might also be tips on how to reduce the chance of infection. Tweets like these are heartwarming as people could be trying to reduce the rate of infection to prevent strain on the HCWs.

## Study 2 on impacts of public responses on HCWs

### Materials and methods

#### Participants

The participants were individuals from Singapore’s healthcare industry across different positions who have worked in the industry before, during, and after Phase 1 (1 June 2020–18 June 2020) and 2 (19 June 2020–17 July) of Singapore’s Circuit Breaker. The Singapore Circuit Breaker was designed to combat the spread of the COVID-19 pandemic within Singapore (Lee, 2020). An HCW is defined as an individual who is currently working in the healthcare industry and providing care and services to patients through direct or indirect means. Direct includes doctors and nurses, while indirect includes aides, helpers, laboratory technicians, or even medical waste handlers (Joseph and Joseph, 2016).

Eighteen participants who completed the interviews included those who were directly (e.g., doctors, nurses, clinic assistants, etc.) and indirectly (e.g., patient service associates) delivering care to patients. The age range of the participants ranges from 21 to 60 years old. Participants are both local Singaporeans and non-locals from China and the Philippines who are working in Singapore. There was no specification on the nationality ratio, and the participants were recruited voluntarily. The detailed participant demographics are provided in Table 1.

**Table 1:** Participant demographics (*N* = 18).

Item	Category	Frequency
Age range	18–24 years old	1
	25–34 years old	8
	35–44 years old	3
	Unknown	6
Gender	Male	4
	Female	14
Nationality	Singaporean	10
	People’s Republic of China (PRC)	6
	Philippine	2
Marital status	Married	4
	Single	9
	Unknown	5
Children	Yes	2
	No	10
	Unknown	6
Working years in the healthcare industry	10 years or more	8
	2 to 5 years	6
	6 to 9 years	3
Occupation	Doctor	1
	Doctor trainee	1
	Nurse clinician	1
	Registered nurse	4
	Senior occupational therapist	1
	Senior patient service associate	1
	Senior staff nurse	3
	Staff nurse	4
	Hospital staff	1
	Vaccination center nurse	1

#### Procedure

We advertised our research study through social networking platforms such as Facebook, Instagram, and LinkedIn and through personal contacts, and asked for individuals from the healthcare sector to participate in interviews. Interested participants were invited to an online interview *via* the Zoom platform with their identities kept private and confidential. Each interview lasted between 30 and 90 min, depending on participants’ experiences and willingness to share their experiences. Of the 18 interviews we conducted, three research assistants first conducted 12 interviews from March to April 2021. The second round of interviews took place in August 2021, where three research assistants subsequently joined and conducted six interviews until the data was saturated. Among all the researchers and research assistants, there was no HCW. According to Khawand and Zargar’s qualitative research (2022), we avoided any presumptions and biases for the purpose of reflexivity. In this case, the influence of the researchers’ personal presumptions was minimized in the process of interviews (Fischer, 2009). When the research assistants conducted interviews, the first and third authors monitored each interview to ensure that dialogs of good quality were established.

After their collection of interviews, the analysis gathered from the first round of interviews in April 2021 was shared with the three research assistants to discuss whether the interpretation and analysis were aligned. All 18 interviews were recorded using the Zoom recording function with participants’ consent. All these recordings were deleted after transcription in MAXQDA software. All designed questions for the interview were asked. The semi-structured interview with open-ended questions was displayed on a slide *via* the screen-sharing function on Zoom for participants to refer to and to minimize potential mishearing and misunderstanding of the question.

The questions used during the interviews have been specially created for the participants to share their experiences for our research objectives. We had four subsets of questions. The first subset was about healthcare workers’ motivation to join or stay in the healthcare industry, e.g., “What motivated you to take this job?” “What are the most important tangible or intangible rewards that you expect in your career?”

The second subset was about general factors that impact their work engagement, e.g., “what positively and what negatively impacted your work engagement and well-being during the COVID-19 pandemic?”

The third subset was about the public views “Does the public view affect your work engagement and well-being as an HCW? In what way?”

The fourth subset was about their HRM practices. Sample questions include “Were there any HR practices or initiatives implemented that were helpful in increasing (or decreasing) your work engagement and well-being during the COVID-19 pandemic? Why and why not?” “Were there any HR practices or initiatives that you wish could be implemented? If not, what would improve your work engagement and well-being during the COVID-19 pandemic?”

The questions about work engagement and well-being were all asked separately. In addition, a set of Chinese-translated interview questions, introduction, explanation, and definitions were done and used during the interviewing of the Chinese-speaking participants. The purpose of the translation is to help those speaking in Chinese to conduct the interview comfortably in their mother language. This enables us to collect more in-depth and high-quality information for the study.

#### Analysis

The first and third authors separately and collectively read through the transcripts, and the codes developed. We triangulated the data as we conducted analysis through discussion between the first and third authors. When assigning codes to each response to each question and developing themes based on the codes, the first and third authors and their research assistants discussed and negotiated various aspects of data and reached a unified conclusion regarding coding and interpretation of the data. During this stage, we had high values of intercoder reliability (0.96), and thus it justified the choice of a single and final coder instead of two or more coders using one set of codes (Burla et al., 2008; Campbell et al., 2013). As a result, with a deep knowledge of the subject matter, the first author coded all the interview transcriptions in MAXQDA Analytics Pro 2020, a software for the analysis of qualitative data (Kuckartz and Rädiker, 2019). During the coding process, the authors kept discussing all the aspects and kept checking the codes and themes to ensure that the results were properly reported.

The transcripts were coded by the question set. We first coded the questions regarding the interviewees’ motivators for them to join and stay in their jobs and the motivators to engage in their jobs during the COVID-19 pandemic. Based on this code, we categorized the calling-motivated interviewees and the extrinsic-motivated interviewees. We then coded what public responses the interviewees received and how they impacted their work engagement and well-being. Finally, we coded what HRM practices or initiatives were effective and ineffective in navigating such impacts. We coded the themes under questions regarding work engagement and well-being separately.

### Results

This qualitative study addressed the major research question: How and why did public responses impact HCWs’ work engagement and well-being? To improve their work engagement and well-being in this area, what did HRM do, and what could HRM do? Findings for these questions were crucial to understanding how to deal with the public responses to better improve the work engagement and well-being levels of HCWs, especially during pandemic crises.

#### How negative public responses impact HCWs’ work engagement and well-being

When asked about the factors that negatively impacted their work engagement and well-being, as well as if and how the public view affected their work engagement, among the 18 interviewees, 9 (50.0%) mentioned that negative public responses negatively impacted their work engagement and 2 (1.8%) mentioned that negative public responses negatively impacted their well-being. Some examples of negative impacts experienced by the HCW’s are illustrated below.

*HCW 14*:“I see a lot of stuff that’s coming out, blaming the nurses lately. I think that me and my friends are quite angry. That’s discrimination on healthcare professionals in general. You can feel like the discrimination is there, there’s a stereotype there. It makes you feel like what’s the point of working so hard people do not even appreciate us.”

*HCW 15*: “When I hear colleagues, my colleagues, when they are being discriminated, that really for me, I feel a sense of anger.”

The “stuff” that HCW14 was referring to was the public responses portrayed in the media and news. Based on HCW 14 and HCW 15, discrimination, blaming of the nurses, and stereotypes are the reality they face in association with their work. Because of the negative public responses, they felt negative emotions, such as anger and unappreciated, which compromise one’s well-being at work. This is consistent with the findings of Study 1.

Specifically, they received negative language responses like criticism, for example:

*HCW 1*: “We go to work, we are also risking our lives to face patients, and we do not know if the patient has or does not have [the virus]; everyone is questioning each other. Do you have COVID? We risk our life, but in a way, we were like, outcast by the public. Like, ‘You work in a hospital. You have COVID.’ That kind of thing. So, I do not have the motivation to go to work. I’m not being appreciated. So, what for?”

*HCW 2*:“By reading some newspapers, I think there’s something happening like that, which made me more alert to the surroundings. I have not been wearing uniform to and from work, so I will continue not to wear it. Yeah.”

Based on the two responses from HCW 1 and HCW 2, the uncertainty about the virus, its effect, and about who has contracted the virus brought additional stress at work, which reflects the finding in Study 1. The public may also associate whoever working in hospitals (i.e., wearing hospital uniforms) were suspected of carrying the virus, and as a result, people would stay away from HCWs or show negative attitudes toward HCWs.

The following cases show a more severe case of physical ostracism, for example:

*HCW 7*: “I have one neighbor. When he heard that I work in a hospital, he stepped back. Then he asked if I worked with COVID patients. But I told him that no, I worked in operation theatre. Then he was like okay already.”

*HCW 3*:“I actually recalled one incident. I was working at the dormitory and the chalet. So, I was coming out from the chalet, then my Grab driver, he asked me, if this place was a quarantine facility? So, I said yes, he rolled down the windows, and he drove at 120/Km per hour. Okay, so I got pissed off, so if you do not want me to sit in your car and you think I’m dirty, you can just tell me. I can leave the car. You do not need to do these kinds of things to me.”

The negative public response ended up restricting the HCWs’ daily living that they used to enjoy before the COVD-19 pandemic. For instance,

*HCW 4*:“When we order food or beverages, if they see the order is from our hospital, they will not take our orders.”

*HCW 1*:“We cannot go on a cruise; we are not allowed to. We have to ask for permission before going on a cruise.”

Furthermore, up to 10 HCWs (55.6%) also faced tough patients and their families who were worried, impatient and did not understand their situations. The following stories revealed that HCWs also needed to face the negative responses from patients’ families, who were worried due to the uncertainty and the inconveniences caused by COVID-19 restrictions.

*HCW 4*:“Since the pandemic, we had a rule that only one caregiver can accompany one patient. Some parents do not understand and then they will abuse us by saying ‘this is stupid;’ ‘why are you so persistent.’ They will have conflicts with us and gave us their bad attitudes.”

*HCW 3*:“There was an influx of very tough patients to handle. So, it affected everyone’s morale….The patients are getting more and more disturbed because they are affected by the pandemic.”

*HCW 1*:“The challenge for me to approach them was so difficult due to social distancing rules. And then, you know, when you do not have face-to-face interaction, sometimes it’s hard for patients to understand you.”

In addition, the following response showed that HCWs were rather limited in explaining their medical practices and procedures to the public, which also fueled the uncertainty that the public faced.

*HCW 8*:“During Phase 1, because I wasn’t in the COVID ward. So initially, I wore my uniform to work. But everyone shun from me. So, I decided not to wear my uniform to work. I can understand that everyone is panicking and anxious, and fear that nurses might have bacterial or nurses and result in spreading. I felt that it has affected my work engagement. Because as a doctor or nurses, we are here to help. We worked with precautious and with PPE very well. But for public they do not know how we are working in the hospital, how careful are we at work. But I cannot explain to people that I am not working in COVID wards. But inside my heart, I would feel a little uncomfortable.”

The above responses from HCWs showed that not only they faced negative responses from the public because of their professions (i.e., wearing uniforms, showing that they work in hospitals), but they were also restricted in their daily living, in presenting their profession to the public, and even in engaging and explaining their professional practices to the public. The negative emotions experienced at work compromised their work engagement and well-being. Because of their professions, HCWs’ receiving negative responses and being restricted in their daily living would bring them to question their calling and purpose at work.

#### Impacts of positive public responses

When asked about what factors positively impacted their work engagement and how public views affected their work engagement, 9 (50.0%) HCWs mentioned that the positive responses from the public positively affected their work engagement, and 2 HCWs (1.8%) mentioned that the positive responses from the public positively improved their well-being. They received positive public responses in terms of encouragement and recognition, for example:

*HCW 6*:“Especially during Phase One, I still remember, almost every week, the words of encouragement, the appreciation letter from the public….Also, encourage letters and encourage words from the public.”

*HCW 9*:“It is just nice that there is a lot of encouragement from the public and a lot of recognition. It is nice to be appreciated.”

The positive public responses also came in terms of tangible donations like food, beverage, gifts, etc.

*HCW 6*:“In fact, the public, community, almost every week they give us food, and also there were donations as well.”

*HCW 16*:“The public also delivers food such as doughnuts, cookies, biscuits for all HCWs [in the hospital].”

*HCW 12*:“I think the public that I met, fortunately, were quite nice. They did not ostracize. In fact, during the COVID period, [in] the hospital, we receive a lot of gifts from the public. Some of them were like fruit juice, a lot of other things or cupcakes, somebody even gave us vitamin C, a very expensive brand, and face moisturizer.”

#### Why public responses impacted HCWs’ work engagement and well-being

To further analyze why public responses had the above impacts on HCWs’ work engagement and well-being, we coded the questions regarding the interviewees’ motivators behind their joining and staying in their jobs and the motivators to engage their jobs during the COVID-19 pandemic. We found that those who had the calling to help people and a sense of meaningfulness and significance in their healthcare careers tend to be more influenced by public responses, both positively and negatively.

More specifically, in the interviews, when asked about why they joined or stayed in their healthcare jobs, calling to help people and the sense of meaningfulness and significance were mentioned 22 times by 12 interviewees. For example,

*HCW 10*:“I always want to help people in terms of relieving pains and helping families to live better and [have] happier lives. And with that, I concluded that medicine is the right field and children’s health is the right field because children are essentially the center of the family.”

*HCW 11*:“When I was younger, I had an illness and I was quite determined to help and to give back and take care of other people and wanted to help other people. As a result, [I was] being exposed to the healthcare industry when I was much younger. So that has been my passion since and it has always been. So like I said, my passion was to help people. So, in my day-to-day job, I get to do that and I can see the influence that I’m able to have on other people’s life.”

*HCW 4*:“I am motivated to work in the healthcare industry because I feel good doing something that helps people and makes people’s lives easier. I feel that it is important to do something that I think is meaningful to myself and others, which keeps me motivated to work everyday.”

Based on their responses, HCW 10, 11, and 4 all expressed that they chose to work in the healthcare industry because they wanted to help people due to their past personal experience or finding a sense of fulfillment from helping others.

In addition to the 12 interviewees who were categorized as calling-motivated HCWs, there were six interviewees who were categorized as motivated by extrinsic motivators such as monetary compensation, job security, or financial bond. When we compared the calling-motivated group with the extrinsically-motivated group, we found a qualitative difference in terms of the extent of being influenced by public views. For instance, among the six interviewees who were motivated by extrinsic motivators, positive impacts were mentioned three times and negative impacts one time. HCW 13 explicitly said her “motivation is money.” And when asked about how public responses influenced her well-being, she said:

*“The public delivers food such as doughnuts, cookies, biscuits for all healthcare workers.”*

On the contrary, all 12 interviews who were motivated by their calling to help people mentioned that they were influenced by the public responses, which included 14 mentions of positive impacts and 15 mentions of negative impacts. In other words, the ones that were motivated by their calling were more sensitive to both positive and negative public responses. For example, when talking about why become an HCW, a calling-motivated interviewee, HCW 14, said,

*“For me, it’s mainly having a passion in medical science that helps me to find ways to [have] a career to help the society in the future, so, then of course nursing fits this role.”*

Then, when asked about what influenced her work engagement during the COVID-19 pandemic, she said,

*“Some of us feel like, this is my passion or calling, like we want to contribute, but when you see the way the public [viewed] us, everyone can feel like the discrimination is there, there’s a stereotype; it makes you feel like what’s the point of working so hard people do not even appreciate us.”*

HCW 14’s response revealed that the negative public response made her question “the point of working so hard” when what she thought as her passion or calling—helping people—was not appreciated by the public in general.

Similarly, HCW 1 said:

*“What motivates me to continue being in the healthcare industry is that, I’d say, being able to help people and understand how they feel. Then from there, even if there are some scenarios where you cannot help, you can always get external organizations to come in to help them, basically being able to help people it’s what motivates me and also my colleagues.”*

HCW 1 further said the following when being asked if public views impacted their work engagement,

*“Ever since the Circuit Breaker started, everybody had no idea how to fight this infectious disease. So whoever was working in healthcare, would [be] criticized or shunned away. It was quite hard, in the first place. For everyone, there was no appreciation of what we do. So at work, even when people come to visit, there’s a lot of tension with the public. So that’s one of the challenges.”*

Both HCW 14 and HCW 1’s responses revealed that they experienced criticism, discrimination, and being shunned away by the people that they were called to help. Their feelings were also translated into tension at work and among colleagues, as they may have felt nervous, stressed, anxious, uptight, or even experiencing low employee morale. Some of them started to question why they should help these people who did not appreciate them.

At the same time, positive public responses could let the calling-motivated HCWs feel warm-hearted. For example, the HCW 12 said:

*“I joined the healthcare industry, because I want to help people. What motivates me to continue being in the healthcare industry is the fact that I see my patients improve. [The patients] are achieving their goals, they are getting back to their life, they are not distracted by stroke or any other impairments, or they can manage their life conditions better, like the chronic ones, and they are more confident; they do not have to stay at home, they can still come out and be a normal life. So I think those are personal rewards for me my job.”*

The same participant, HCW 12, said the following when being asked about what influenced her work engagement:

*“Generally, I think the public that I met, fortunately, they were quite nice. They did not ostracize. In fact, during the COVID period, [at] the hospital, we receive a lot of gifts from the public. Then some of them they were like fruit juice and a lot of other things or cupcakes, somebody even gave us vitamin C from a very expensive brand. And also like, face moisturizer, cream.*

Therefore, comparing both groups of HCWs, extrinsically-motivated and calling-motivated, we conclude that for those HCWs who were motivated by the calling to help people, the impact of public responses on their work engagement and well-being was more salient.

#### Effective HRM practices regarding public responses

When asked about the HR practices or initiatives implemented that increased their work engagement and well-being during the COVID-19 pandemic, 3 HCWs mentioned that HRM conveying the recognition or appreciation from the public or patients improved their work engagement, and 1 mentioned the improvement of well-being, for example:

*HCW 10*:“They sent a lot of messages from the community to us so that we know that our effort is being recognized.”

*HCW 12*:“They also try to get patients to write ‘thank you’ notes on why they appreciate us.”

There were 24 mentions about HR departments delivering tangible benefits sponsored by the public, which could enhance their work engagement, and nine mentions about such HRM practices would improve their well-being. These tangible benefits included free food catering, vouchers or discounts, staycation, or other forms of donations sponsored by the public, for example:

*HCW 10*:“And they did organize a lot of things for us, for example, all those retreats, and some of them are sponsored by the community and the previous patients. So the hospital passes on all the messages to us so that everybody knows that our work is being appreciated by the community.”

*HCW 7*:“Got one hotel staycation, but I think it was given out by other organizations, not by our hospital. But it’s a free staycation for healthcare workers….I think from the public, when they donate things to us, the hospital will pass it on to different wards in rotations. They also gave us things like skin lotion and shower gel.”

*HCW 2*:“Maybe food? We’ve got a lot of sponsors. And I’m actually quite grateful for the sponsors. I think the HR did a good job in maintaining the pantry with food. There’s always food. You can get the food whenever you want to.”

*HCW 1*: They do give us bentos. They were also Grab vouchers for us, both food and transport….They do give us a lot of support when they come up with these vouchers.”

*HCW 6*:“Every week, we receive food from the public and the community as a kind of encouragement.”

*HCW 17*:“Healthcare packs: when we receive, we will feel that the company is thinking for us, and we will feel appreciated. Everyone likes to receive gifts.”

*HCW 5*:“Providing us with food catering for pandemic wards, Grab or taxi voucher, COVID-19 badge, Nurse Day hamper, Corporate pass for tourist attraction places. (They were) stopped for a few years already, for example, free entry to the zoo and bird park for up to 4 headcounts.”

The above quotes showed that their respective HR departments or equivalents managed the rewards and benefits for HCWs, including distribution of donations, replenishing the pantry, and providing food catering or vouchers. This type of practice implies an active role of HR practitioners in providing support and care for HCWs, so that HCWs “would feel appreciated.”

#### HRM practices regarding public responses in hope

For the question about “what are the HR practices or initiatives that they wish could be implemented but not that would improve their work engagement and well-being during the COVID-19 pandemic,” five mentions were that they would welcome more tangible benefits such as food delivery and discount, for example:

*HCW 1*:“I think it’s really cool. So they can actually offer a monthly food package where we just pay a certain amount and settle for us, and it delivers, or you can either collect it somewhere accessible by staff, or they can send it to our work desk. We have a service internally that allows delivery with robots. So I think there’s one good initiative that they can do. We have robots, you know; you do not need a person.”

*HCW 5*:“More benefits, probably. For example, more pertaining to family, like enrichment programs or family discounts for tourist attractions.”

The reason why HCWs would appreciate bento’s is because at the peak of the COVID-19 pandemic, they had to sleep and eat in hospitals for many days before they changed shifts. They would probably eat food from their respective cafeteria. Therefore, having outside food delivered to them would be a reward so that they could take a break from the usual cafeteria food and enjoy a moment of bliss outside of the hospital.

At the same time, it is noteworthy that 2 of the mentions indicated that they needed such benefits because of the social ostracism in public.

*HCW 1*:“During Phase 2 to 3, where we were allowed to gather in bigger groups. It’s okay to go out in groups to settle our own food. But for us, we are still getting the stares. We would prefer a place, where it’s just all healthcare staff. I understand that they are worried that they might contract COVID by just being near us. So, if the bento continues, this can help us.”

*HCW 1*:“The bento stopped after a while. We can continue that. I mean, if we can, you know, instead of going to the public, even though it’s common now, but people do still like you know, there is still a case. You do not know where they have been, you know, they are still cautious. With us, even though it’s in a hospital, and I mean, you come and visit you are really committed. So I think they got one by which they continue the initiative. I do not mind paying.”

#### Ineffective HRM practices regarding public responses

For the question about what HR practices had decreased their work engagement during the COVID-19 pandemic, 1 HCW mentioned that HRM provided too few tangible benefits to the public. For instance,

*HCW 3*:“Like sometimes they give us little snacks, vouchers but, do these vouchers make a difference? We are still very drained, right.”

To some HCWs, while they may appreciate the tangible benefits, such as snacks and vouchers, their working conditions have not changed; they would also be too physically exhausted or tied up with long hours to enjoy the tangible reward.

Unexpectedly, three HCWs mentioned that they were unsure if it was from HRM.

*HCW 8*:“There’s a hotel staycation, free for HCWs, but I am not sure if it’s the hospital or other people give us.”

*HCW 12*:“I’m not sure whether it was the HR department, but we definitely receive a lot of anonymous gifts like sponsorships such as all the different food that comes in. So I guess, there is the food and water. I’m not sure whether it’s like the HR or it could be just my manager.”

*HCW 2*:“I’m confused. Who is doing all this? Actually. I do not know if it is the HR or is it the government?”

As shown in these responses from HCWs, they were not clear whether the delivery of the tangible reward was from their respective HR departments or from other organizations. There could be a few reasons attributed to the doubt about HR’s role in supporting their work engagement. First, the HR department in the respective hospitals was not present or active in providing support. Second, communication from HR departments could be lacking, and the HR managers may not be “on the ground” with the HCWs. Third, the organization structure of the hospital could be complex in that HCWs being in the frontline would not normally interact with backend administrative support. This was probably because HRM was far away physically (occurrence frequency = 6) or HRM lacked communication or interaction with them due to working from home (occurrence frequency = 12), for example:

*HCW 7*:“I feel that HR are very far away from us. Our hospital is very big, and I do not even know who is in the HR department.”

*HCW 11*:“When I feel that they are not concerned for me, it builds up this antagonizing relationship….They do not have a strong presence in my working life.”

*HCW 2*:“HR? More like the managers. Okay. Love and belonging. I do not think HR will go that far.”

*HCW 9*:“However, the HR left the HOD to manage us overall. This is because I think they have a manpower issue to help us personally.”

*HCW 1*:“HR does not care about my needs during the COVID-19 pandemic. Not really. It’s during the pandemic, so there’s minimized interaction with one another. So it’s very hard for them to also meet our needs….”

*HCW 5*:“I think it is so difficult to interact deeply with HR.”

## Discussion

Two studies with mixed methods demonstrate that mixed public responses toward HCWs have impacted their work engagement and well-being differently, and HRM could do something effective to intervene. Study 1 uncovers the mixed public responses toward HCWs existed online, with over half (55.9%) of positive emotions, 27.2% neutral, and 16.9% negative emotions. The results of topic modeling on each sentiment indicated that the commonly identified topics were related to their emotional fear, uncertainty, rejection of the negative sentiment data, and appreciation or gratitude for the positive sentiment data.

While Study 1 captured what kind of mixed responses toward HCWs generally existed in the online public environment from the perspective of the public itself. Study 2 was designed to explore the perspective of HCWs to uncover how and why such responses impacted HCWs. Specifically, Study 2 further uncovered the more specific manifestations of the public’s fear and appreciation as identified in Study 1, and revealed how and why these mixed public responses impacted HCWs’ work engagement and well-being. In Study 2, we also explored how HRM could intervene or increase the capacities or resources of HCW to better deal with these public responses.

The results show that negative public responses were manifested as criticism, physical ostracism, rejection, and discrimination, and such responses compromised HCWs’ work engagement and well-being. On the other hand, positive public responses were manifested as encouragement, recognition and tangible donations, and such responses improved HCWs’ work engagement and well-being. Furthermore, these mixed impacts were more salient among those with an occupational calling to help others as compared to those driven by extrinsic motivators like money. To moderate such impacts, HRM, who helped convey the recognition or appreciation from the public or deliver tangible benefits sponsored by the public, improved HCWs’ work engagement and well-being. HCWs would also expect the human resource department to cater food for them due to social ostracism in public. On the other hand, HRM who failed to do these or were absent in communication with HCWs decreased their work engagement and well-being.

Our results are consistent with the Self-Determination Theory, which proposes that when the needs for competency, relatedness, and autonomy are satisfied by the social environments, individuals will harvest good work engagement and well-being. An occupational calling provides meaning and enjoyment in an HCW’s life and becomes part of an HCW’s integrated sense of self. Therefore, when public responses are consistent with their integrated sense of self, they would facilitate satisfaction of the three needs. Consistently, our results suggest that the impacts of public responses on work engagement and well-being were more salient among HCWs who had an occupational calling than those without an occupational calling. On the other hand, the work engagement and well-being of those who do not have a calling to their healthcare vocation might depend on whether those three needs are met in other domains.

### Implications for practice

The findings from our studies first serve as an alarm for the public about how they talk about or treat HCWs during health crises in various settings. Because public responses do impose an impact on HCWs in terms of their work engagement and well-being, the public should have the responsibility to respond properly. For example, the public should remind themselves of the observable and hidden consequences when they plan to post negative responses on social media. To navigate such public responses, various institutions such as the government, mass media, and WHO must take effective actions to help reduce the uncertainties and fear about COVID-19. To reduce the negative responses toward HCWs, active management efforts can be taken, such as proper health education targeting the public (Bagcchi, 2020). On the other hand, the government and mass media should also create an environment and atmosphere of appreciation and recognition for the sacrifice of HCWs, which are suggested to increase their work engagement and well-being.

Our research further provides insight into planning effective HRM initiatives and implementing effective HRM practices in response to the public views or reactions toward HCWs. Our results suggest that, to improve HCWs’ work engagement and well-being, HRM professionals could serve as the bridge between positive public responses and HCWs by passing encouragement and recognition from the public and by distributing tangible benefits sponsored by the public. We recommend HRM practitioners in the healthcare industry consider creating a culture that supports nursing staff and providing them with a well-balanced total reward system that is flexible and adjustable, particularly at the time of crisis. For example, they can proactively contact relevant companies and governmental agencies to donate various benefits like discounts, vouchers, foods, and gifts, to the HCWs. HRM professionals can also proactively organize activities among communities and patients to convey verbal encouragement and recognition for HCWs in different terms. Specifically, to recognize the efforts of HCWs, HRM can post appreciation letters from the public or patients at the workplace of HCWs, or organize online events or letter-writing campaigns to gather encouraging words from the public and patients and deliver them to HCWs. We also recommend HRM executives in the healthcare industry must be more active in their communication with HCWs. More specifically, better communication channels that suit frontline workers who do not have access to phones and news as easily are recommended. Although HRM professionals may be working from home due to the pandemic, proactive communication and organized support would show that the organization is compassionate toward HCWs’ efforts. HRM practitioners also need to develop its own presence and branding within the organization so that the HR department would be recognized and value add their initiatives. To tackle the issues incurred by the negative responses, the HRM department can initiate counseling, workshops, or hotlines to educate HCWs on how to react in a positive way.

### Strengths, limitations, and future directions

In this paper, we have explored a new approach to understanding public responses toward HCWs during the pandemic, revealed how and why they impacted HCWs’ work engagement and well-being, and provided practical recommendations for HRM professionals to better navigate these impacts. Our research has opened many interesting areas to extend the research in the future. For Study 1, first, we are currently utilizing only Twitter data. Additional data from social media platforms such as Facebook and Instagram, which allow for more words and images, would also provide more interesting insights than Twitter which contains word limits. Second, we use the predefined set of hashtags to identify those comments toward HCWs in Twitter data. There are more tweets without these hashtags that could potentially relate to HCWs as well. It will be interesting to explore more advanced event and topic detection algorithms such as sarcasm detection to discover more interesting comments from Twitter or other social media platforms.

For Study 2, our sample size of 18 was relatively small, even though the interviews provided deep insights on the topic. Future studies could use survey techniques to expand the number and occupational categories to a broader range to account for all areas of the healthcare industry, ensuring more representative findings and analysis. In addition, survey can also help identify the quantitative predictions of public responses on HCWs’ work engagement and well-being. As our sample for Study 2 is restricted, future investigations may benefit from including samples from various areas of the healthcare industry and human resources, for example, specialists. In this way, scholars could map the potential consequences of COVID-19 in various areas and institutions.

Additionally, due to safe-distancing measures during the COVID-19 pandemic, on-site face-to-face interviews were avoided for our data collection. Instead, the interviews were conducted *via* Zoom, and the camera function was also turned off to protect the identity of the participants. Therefore, this limited the ability to discern the participants’ non-verbal cues during the interviews, which could have provided greater insights as such cues would have complemented the actual words that were spoken and added to their meaning. Future research can consider face-to-face physical interviews that allow the analysis of their body language to better interpret their answers.

Lastly, the current sample of interviewees worked in various sizes of hospitals and clinics, which suggest that the capability of HRM would vary across organizations. Future research can also incorporate the use of interviews and surveys from HR professionals in the healthcare sector to gain a deeper understanding of HRM strategy and planning.

## Conclusion

Our findings highlight the significant impact of public responses on HCWs’ work engagement and well-being and what HRM professionals could do to navigate these impacts. Although many of the online sentiments are positive (i.e., gratitude, caring, including appreciations and positive wishes) and some are negative (i.e., emotional uncertainty) toward HCWs during the pandemic, it is noteworthy that both existing positive and negative public responses could impact HCWs’ work engagement and well-being. Specifically, negative public responses affected HCWs’ work engagement and well-being negatively, and positive public responses impacted them positively. Furthermore, such differentiated impacts were more salient among the HCWs who were motivated by calling as compared to those who were motivated by extrinsic factors. As such, our research contributes to the literature by suggesting self-determination theory and vocational calling as a mechanism to explain why public responses could exert such impacts. Based on our results, to promote the positive influence of positive public responses, we call for HRM professionals to serve as a bridge by proactively conveying verbal recognition and passing tangible donations from the public. Moreover, to prevent compromising of HCWs’ work engagement and well-being, HRM professionals should make themselves visible or present despite the work-from-home policy through proactive communication with HCWs, highlighting their initiatives, and communicating with HCWs. We believe this is of great importance for handling crises in the healthcare industry in the future.

## Data availability statement

The raw data supporting the conclusions of this article will be made available by the authors, without undue reservation.

## Ethics statement

The studies involving human participants were reviewed and approved by the Singapore University of Social Sciences IRB Committee. The patients/participants provided their written informed consent to participate in this study.

## Author contributions

WS was responsible for the overall research design and data collection and analysis of Study 2. ZW was responsible for the data collection and analysis of Study 1. MS was responsible for the theory development and data collection of Study 2. WS and ZW wrote the first draft of the manuscript. All authors contributed to the article and approved the submitted version.

## Conflict of interest

The authors declare that the research was conducted in the absence of any commercial or financial relationships that could be construed as a potential conflict of interest.

## Publisher’s note

All claims expressed in this article are solely those of the authors and do not necessarily represent those of their affiliated organizations, or those of the publisher, the editors and the reviewers. Any product that may be evaluated in this article, or claim that may be made by its manufacturer, is not guaranteed or endorsed by the publisher.

## Footnotes

^1^ https://www.nltk.org/

^2^ https://pypi.org/project/textblob/0.9.0/

^3^ https://pyldavis.readthedocs.io/en/latest/

## References

[ref1] Akef I. Arango J. S. M. Xu X. (2016). Mallet vs. GenSim: topic modeling for 20 news groups report. Univ. Ark. Little Rock Law J. 2:39205. doi: 10.13140/RG.2.2.19179.39205/1

[ref2] Alfes K. Shantz A. Truss C. (2012). The link between perceived HRM practices, performance and well-being: the moderating effect of trust in the employer. Hum. Resour. Manag. J. 22, 409–427. doi: 10.1111/1748-8583.12005

[ref3] Allande-Cussó R. García-Iglesias J. J. Ruiz-Frutos C. Domínguez-Salas S. Rodríguez-Domínguez C. Gómez-Salgado J. (2021). Work engagement in nurses during the Covid-19 pandemic: A cross-sectional study. Healthcare 9:253. doi: 10.3390/healthcare9030253 33804351 PMC8001401

[ref4] Allen T. (2020). 7 facts on essential workers Hazard pay that you need to know now [online]. Forbes. Available at: https://www.forbes.com/sites/terinaallen/2020/05/20/7-facts-on-essential-workershazard-pay-that-you-need-to-know-now/#466528541483 (Accessed September 2022).

[ref5] Anwar K. Qadir G. H. (2017). A study of the relationship between work engagement and job satisfaction in private companies in Kurdistan. Int. J. Adv. Eng. Manage. Sci. 3, 1102–1110. doi: 10.24001/ijaems.3.12.3

[ref6] Bagcchi S. (2020). Stigma during the COVID-19 pandemic. Lancet Infect. Dis. 20:782. doi: 10.1016/S1473-3099(20)30498-9, PMID: 32592670 PMC7314449

[ref7] Barello S. Palamenghi L. Graffigna G. (2020). Stressors and resources for healthcare professionals during the Covid-19 pandemic: lesson learned from Italy. Front. Psychol. 11:2179. doi: 10.3389/fpsyg.2020.02179, PMID: 33117208 PMC7578218

[ref8] Berg J. M. Grant A. M. Johnson V. (2010). When callings are calling: crafting work and leisure in pursuit of unanswered occupational callings. Organ. Sci. 21, 973–994. doi: 10.1287/orsc.1090.0497

[ref9] Booth W. Adam K. Rolfe P. (2020). In fight against coronavirus, the world gives medical heroes a standing ovation. [online]. Washington post. Available at: https://www.washingtonpost.com/world/europe/clap-for-carers/2020/03/26/3d05eb9c6f66-11ea-a156-0048b62cdb51_story.html (Accessed September 2022).

[ref10] Broeck A. Vansteenkiste M. Witte H. Soenens B. Lens W. (2010). Capturing autonomy, competence, and relatedness at work: construction and initial validation of the work-related basic need satisfaction scale. J. Occup. Organ. Psychol. 83, 981–1002. doi: 10.1348/096317909X481382

[ref11] Burla L. Birte K. Jurgen B. Katharina L. Margreet D. Thomas A. (2008). From text to Codings: inter-coder reliability assessment in qualitative content analysis. Nurs. Res. 57, 113–117. doi: 10.1097/01.NNR.0000313482.33917.7d, PMID: 18347483

[ref12] Campbell E. Popescu G. H. (2021). Psychological distress, moral trauma, and burnout syndrome among COVID-19 frontline medical personnel. Psychosociol. Issues Hum. Resour. Manag. 9, 63–76. doi: 10.22381/pihrm9220215

[ref13] Campbell J. L. Quincy C. Osserman J. Pedersen O. K. (2013). Coding in-depth semi-structured interviews: problems of unitization and intercoder reliability and agreement. Sociol. Methods Res. 42, 294–320. doi: 10.1177/0049124113500475

[ref14] Christian M. S. Garza A. S. Slaughter J. E. (2011). Work engagement: a quantitative review and test of its relations with task and contextual performance. Pers. Psychol. 64, 89–136. doi: 10.1111/j.1744-6570.2010.01203.x

[ref15] Cohen S. Nica E. (2021). COVID-19 pandemic-related emotional anxiety, perceived risk of infection, and acute depression among primary care providers. Psychosociol. Issues Hum. Resour. Manag. 9, 7–20. doi: 10.22381/pihrm9220211

[ref16] Creed P. A. Rogers M. E. Praskova A. Searle J. (2014). Career calling as a personal resource moderator between environmental demands and burnout in Australian junior doctors. J. Career Dev. 41, 547–561. doi: 10.1177/0894845313520493

[ref17] Denning M. Goh E. T. Tan B. Kanneganti A. Almonte M. Scott A.. (2021). Determinants of burnout and other aspects of psychological well-being in healthcare workers during the COVID-19 pandemic: a multinational cross-sectional study. PLoS One 16:e0238666. doi: 10.1371/journal.pone.0238666, PMID: 33861739 PMC8051812

[ref18] Dik B. J. Duffy R. D. (2009). Calling and vocation at work. Couns. Psychol. 37, 424–450. doi: 10.1177/0011000008316430

[ref19] Duffy R. D. Dik B. J. Steger M. F. (2011). Calling and work-related outcomes: career commitment as a mediator. J. Vocat. Behav. 78, 210–218. doi: 10.1016/j.jvb.2010.09.013

[ref20] Dye T. D. Alcantara L. Siddiqi S. Barbosu M. Sharma S. Panko T.. (2020). Risk of COVID-19-related bullying, harassment and stigma among healthcare workers: an analytical cross-sectional global study. BMJ Open 10:e046620. doi: 10.1136/bmjopen-2020-046620, PMID: 33380488 PMC7780430

[ref21] Elangovan A. R. Pinder C. C. McLean M. (2010). Callings and organizational behaviour. J. Vocat. Behav. 76, 428–440. doi: 10.1016/j.jvb.2009.10.009

[ref22] Fischer C. T. (2009). Bracketing in qualitative research: conceptual and practical matters. Psychother. Res. 19, 583–590. doi: 10.1080/10503300902798375, PMID: 20183407

[ref23] Gagné M. Deci E. L. (2005). Self-determination theory and work motivation. J. Organ. Behav. 26, 331–362. doi: 10.1002/job.322

[ref24] Garfin D. R. Silver R. C. Holman E. A. (2020). The novel coronavirus (COVID-2019) outbreak: amplification of public health consequences by media exposure. Health Psychol. 39, 355–357. doi: 10.1037/hea0000875, PMID: 32202824 PMC7735659

[ref25] Gazica M. W. Spector P. E. (2015). A comparison of individuals with unanswered callings to those with no calling at all. J. Vocat. Behav. 91, 1–10. doi: 10.1016/j.jvb.2015.08.008

[ref26] Gómez-Salgado J. Domínguez-Salas S. Romero-Martín M. Romero A. Coronado-Vázquez V. Ruiz-Frutos C. (2021). Work engagement and psychological distress of health professionals during the COVID-19 pandemic. J. Nurs. Manag. 29, 1016–1025. doi: 10.1111/jonm.13239, PMID: 33400325

[ref27] González-Romá V. Schaufeli W. B. Bakker A. B. Lloret S. (2006). Burnout and work engagement: independent factors or opposite poles? J. Vocat. Behav. 68, 165–174. doi: 10.1016/j.jvb.2005.01.003

[ref28] Gordon S. Nelson S. (2005). An end to angels: moving away from the ‘virtue script’ toward a knowledge-based identity for nurses. Am. J. Nurs. 105, 62–69. doi: 10.1097/00000446-200505000-00031, PMID: 15867536

[ref29] Grey I. Arora T. Thomas J. Saneh A. Tohme P. Abi-Habib R. (2020). The role of perceived social support on depression and sleep during the COVID-19 pandemic. Psychiatry Res. 293:113452. doi: 10.1016/j.psychres.2020.113452, PMID: 32977047 PMC7500407

[ref30] Harter J. K. Schmidt F. L. Hayes T. L. (2002). Business-unit-level relationship between employee satisfaction, employee engagement, and business outcomes: a meta-analysis. J. Appl. Psychol. 87, 268–279. doi: 10.1037/0021-9010.87.2.268, PMID: 12002955

[ref31] Hessekiel D. (2020). Creative ways companies are giving Back during the COVID-19 crisis [online]. Forbes Available at: https://www.forbes.com/sites/davidhessekiel/2020/03/27/creative-ways-companies-are-giving-back-during-the-covid-19-crisis/?sh=37c9dee07f14 (Accessed September 2022).

[ref32] Hirschi A. (2012). Callings and work engagement: moderated mediation model of work meaningfulness, occupational identity, and occupational self-efficacy. J. Couns. Psychol. 59, 479–485. doi: 10.1037/a0028949, PMID: 22774870

[ref33] Hwang J. Yong E. Cheong K. Ling Z. J. Goh L. H. Lim F. S.. (2020). Responding to the COVID-19 pandemic: the role of occupational health services in a tertiary hospital in Singapore. J. Occup. Health 62:e12172. doi: 10.1002/1348-9585.12172, PMID: 33058404 PMC7557359

[ref34] IFMSA (2020). Declaration by the health Care in Danger Community of concern about the current situation of violence against health care [online]. IFMSA, Joint Communication.

[ref35] Joseph B. Joseph M. (2016). The health of the healthcare workers. Indian J. Occup. Env. Med. 20, 71–72. doi: 10.4103/0019-5278.197518, PMID: 28194078 PMC5299814

[ref36] Khanal P. Devkota N. Dahal M. Paudel K. Joshi D. (2020). Mental health impacts among health workers during COVID-19 in a low resource setting: a cross-sectional survey from Nepal. Glob. Health 16, 1–12. doi: 10.1186/s12992-020-00621-z PMC7517059 32977818

[ref01] Khawand S. Zargar P. (2022). Job autonomy and work-life conflict: A conceptual analysis of teachers’ wellbeing during COVID-19 pandemic. Front Psychol. 13:882848. doi: 10.3389/fpsyg.2022.882848, PMID: 35959051 PMC9359983

[ref37] Koh D. Lim M. K. Chia S. E. Ko S. M. Qian F. Ng V.. (2005). Risk perception and impact of severe acute respiratory syndrome (SARS) on work and personal lives of healthcare Workers in Singapore What can we learn? Med. Care 43, 676–682. doi: 10.1097/01.mlr.0000167181.36730.cc, PMID: 15970782

[ref38] Kuckartz U. Rädiker S. (2019). Analyzing qualitative data with MAXQDA. Cham: Springer.

[ref39] Lee H. L. (2020). PM Lee: the COVID-19 situation in Singapore (3 Apr) [online]. A Singapore government agency website. Available at: https://www.gov.sg/article/pm-lee-hsien-loong-on-the-covid-19-situation-in-singapore-3-apr (Accessed September 2022).

[ref40] Lum A. Goh Y. L. Wong K. S. Seah J. Teo G. Ng J. Q.. (2021). Impact of COVID-19 on the mental health of Singaporean GPs: a cross-sectional study. BJGP Open. 5:BJGPO.2021.0072. doi: 10.3399/BJGPO.2021.0072, PMID: 34172477 PMC8450882

[ref41] Marey-Sarwan I. Hamama-Raz Y. Asadi A. Nakad B. Hamama L. (2022). “It's like we're at war”: nurses’ resilience and coping strategies during the COVID-19 pandemic. Nurs. Inq. 29:e12472. doi: 10.1111/nin.12472, PMID: 34724283 PMC8646746

[ref42] Markos S. Sridevi M. S. (2010). Employee engagement: the key to improving performance. Int. J. Bus. Manage. 5:89. doi: 10.5539/ijbm.v5n12p89

[ref43] Marton G. Vergani L. Mazzocco K. Garassino M. C. Pravettoni G. (2020). 2020s heroes are not fearless: the impact of the COVID-19 pandemic on well-being and emotions of italian health care workers during Italy phase 1. Front. Psychol. 11:2781. doi: 10.3389/fpsyg.2020.588762 PMC7593839 33178088

[ref44] Maxwell S. Grupac M. (2021). Virtual care technologies, wearable health monitoring sensors, and internet of medical things-based smart disease surveillance Systems in the Diagnosis and Treatment of COVID-19 patients. Am. J. Med. Res. 8, 118–131. doi: 10.22381/ajmr8220219

[ref45] Muthuri R. N. Senkubuge F. Hongoro C. (2020). Determinants of motivation among healthcare Workers in the East African Community between 2009–2019: a systematic review. Healthcare 8:164. doi: 10.3390/healthcare8020164, PMID: 32532016 PMC7349547

[ref46] Nemțeanu M. S. Dinu V. Dabija D. C. (2021). Job insecurity, job instability and job satisfaction in the context of COVID-19 pandemic. J. Competitive. 13, 65–82. doi: 10.7441/joc.2021.02.04

[ref47] Nemțeanu M. S. Dinu V. Pop R. A. Dabija D. C. (2022). Predicting job satisfaction and work engagement behavior in the COVID-19 pandemic: a conservation of resources theory approach. E&M Econ. Manage. 25, 23–40. doi: 10.15240/tul/001/2022-2-002

[ref48] Pappa S. Ntella V. Giannakas T. Giannakoulis V. G. Papoutsi E. Katsaounou P. (2020). Prevalence of depression, anxiety, and insomnia among healthcare workers during the COVID-19 pandemic: a systematic review and meta-analysis. Brain Behav. Immun. 88, 901–907. doi: 10.1016/j.bbi.2020.05.026, PMID: 32437915 PMC7206431

[ref49] Pradhan R. K. Hati L. (2019). The measurement of Eemployee well-being: Development and validation of a scale. Glob. Bus. Rev. 23, 385–407. doi: 10.1177/0972150919859101

[ref51] Rahul K. Jindal B. R. Singh K. Meel P. (2021). "Analysing public sentiments regarding COVID-19 vaccine on twitter", in: 7th international conference on advanced computing and communication systems (ICACCS): IEEE, 488–493.

[ref52] Riley A. Nica E. (2021). Internet of things-based smart healthcare systems and wireless biomedical sensing devices in monitoring, detection, and prevention of COVID-19. Am. J. Med. Res. 8, 51–64. doi: 10.22381/ajmr8220214

[ref53] Ryan R. M. Deci E. L. (2000). Self-determination theory and the facilitation of intrinsic motivation, social development, and well-being. Am. Psychol. 55, 68–78. doi: 10.1037/0003-066X.55.1.68, PMID: 11392867

[ref54] San Juan N. V. Aceituno D. Djellouli N. Sumray K. Regenold N. Syversen A.. (2021). Mental health and well-being of healthcare workers during the COVID-19 pandemic in the UK: contrasting guidelines with experiences in practice. BJPsych. Open 7. doi: 10.1101/2020.07.21.20156711 PMC7844154 33298229

[ref55] Schaufeli W. B. Salanova M. González-Romá V. Bakker A. B. (2002). The measurement of engagement and burnout: a two sample confirmatory factor analytic approach. J. Happiness Stud. 3, 71–92. doi: 10.1023/A:1015630930326

[ref56] Secker R. Braithwaite E. (2021). Social media induced secondary traumatic stress: can viewing news relating to knife crime via social media induce PTSD symptoms? Psychreg. J. Psychol. 5.

[ref57] Shantz A. Alfes K. Arevshatian L. (2016). HRM in healthcare: the role of work engagement. Pers. Rev. 45, 274–295. doi: 10.1108/PR-09-2014-0203

[ref58] Singh R. Subedi M. (2020). COVID-19 and stigma: social discrimination towards frontline healthcare providers and COVID-19 recovered patients in Nepal. Asian J. Psychiatr. 53:102222. doi: 10.1016/j.ajp.2020.102222, PMID: 32570096 PMC7293527

[ref59] Strömgren M. Eriksson A. Bergman D. Dellve L. (2016). Social capital among healthcare professionals: a prospective study of its importance for job satisfaction, work engagement and engagement in clinical improvements. Int. J. Nurs. Stud. 53, 116–125. doi: 10.1016/j.ijnurstu.2015.07.012, PMID: 26315780

[ref60] Szomszor M. Kostkova P. St Louis C. (2009). "Twitter informatics: tracking and understanding public reaction during the 2009 swine flu pandemic", in: 2011 IEEE/WIC/ACM international conferences on web intelligence and intelligent agent technology, 320–323.

[ref61] Tan B. Y. Chew N. W. Lee G. K. Jing M. Goh Y. Yeo L. L.. (2020a). Psychological impact of the COVID-19 pandemic on health care workers in Singapore. Ann. Intern. Med. 173, 317–320. doi: 10.7326/M20-1083, PMID: 32251513 PMC7143149

[ref62] Tan B. Y. Kanneganti A. Lim L. J. Tan M. Chua Y. X. Tan L.. (2020b). Burnout and associated factors among health care workers in Singapore during the COVID-19 pandemic. J. Am. Med. Dir. Assoc. 21, 1751–1758.e5. e1755. doi: 10.1016/j.jamda.2020.09.035 33256955 PMC7534835

[ref63] Tomo A. De Simone S. (2017). Exploring factors that affect the well-being of healthcare workers. Int. J. Bus. Manage. 12, 49–61. doi: 10.5539/ijbm.v12n6p49

[ref64] Wang Z. Bai G. Chowdhury S. Xu Q. Seow Z. L. (2017). Twiinsight: discovering topics and sentiments from social media datasets. arXiv [Preprint]. arXiv:1705.08094.

[ref65] WHO. (2021). WHO and partners call for action to better protect health and care workers from COVID-19 [online]. Available at: https://www.who.int/news/item/21-10-2021-who-and-partners-call-for-action-to-better-protect-health-and-care-workers-from-covid-19 (Accessed September 2022).

[ref66] Wicker E. (2020). Coronavirus: thanking healthcare workers worldwide [online]. BBC News. Available at: https://www.bbc.com/news/av/world-52536070 (Accessed September 2022).

[ref67] Xiong Y. Peng L. (2020). Focusing on healthcare providers' experiences in the COVID-19 crisis. Lancet Glob. Health 8, e740–e741. doi: 10.1016/S2214-109X(20)30214-X, PMID: 32573442 PMC7190304

[ref68] Zhang M. Zhang P. Liu Y. Wang H. Hu K. Du M. (2021). Influence of perceived stress and workload on work engagement in frontline nurses during COVID-19 pandemic. J. Clin. Nurs. 30, 1584–1595. doi: 10.1111/jocn.15707, PMID: 33590524 PMC8014711

[ref69] Ziedelis A. (2019). Perceived calling and work engagement among nurses. West. J. Nurs. Res. 41, 816–833. doi: 10.1177/0193945918767631, PMID: 29591587

